# Out-of-hospital cardiac arrest incidence and temporal trends in Ecuador: a national cross-sectional study

**DOI:** 10.1016/j.resplu.2026.101467

**Published:** 2026-08-26

**Authors:** Ricardo S. Pinto-Villalba, Mónica Martínez, Carlos Guerrero-Calderón, Jefferson Reinoso, Carla Zamora, Rene M. Abarca-Tenemasa

**Affiliations:** a Universidad UTE, Facultad Ciencias de la Salud Eugenio Espejo, Carrera Atención Prehospitalaria y Emergencias, Grupo de Investigación en Medicina Prehospitalaria, GRIMPE, Quito 170527, Ecuador; b Universidad Central del Ecuador, Carrera de Atención Prehospitalaria, Quito 170403, Ecuador

**Keywords:** Out-of-hospital cardiac arrest, Incidence, Epidemiology, Low- and middle-income countries, Temporal trends

## Abstract

**Background:**

Out-of-hospital cardiac arrest (OHCA) is a leading global cause of mortality, with wide regional disparities in incidence. While high-income countries have national OHCA registries national data from Latin America remain scarce, limiting context-specific emergency planning.

**Objective:**

To describe the incidence and temporal trends of confirmed EMS-attended OHCA in Ecuador from 2022 to 2024, and to explore ecological correlations with cardiovascular and socioeconomic indicators.

**Methods:**

We conducted a retrospective observational study, following STROBE guidelines, using confirmed OHCA cases recorded by the Integrated Security Service (SIS ECU 9-1-1) from 1 January 2022 to 31 December 2024. Temporal and geographic trends were analysed and visualised. Incidence rates were calculated per 100,000 inhabitants, and province-year ecological associations with hypertension, diabetes, acute myocardial infarction and gross value added (GVA) were assessed using Spearman’s rank correlation.

**Results:**

SIS ECU 9-1-1 recorded 936,784 emergency events, of which 9942 (1.06%) were confirmed EMS-attended OHCA cases. National incidence decreased from 20.51 per 100,000 inhabitants in 2022 to 18.71 in 2023 and 16.52 in 2024. Higher adjusted rates were observed in Loja, Imbabura and Carchi, while Guayas showed persistently low rates. OHCA correlated moderately with diabetes (rho = 0.463; *p* < 0.001) and weakly but significantly with acute myocardial infarction (rho = 0.382; *p* < 0.001). Associations with hypertension and GVA were weak and non-significant.

**Conclusions:**

This first national epidemiological profile of OHCA in Ecuador shows declining incidence, geographical heterogeneity and relevant ecological associations with diabetes and acute myocardial infarction. The findings support a national OHCA registry and targeted prevention and emergency-response strategies.

## Introduction

Out-of-hospital cardiac arrest (OHCA) is a major global public health concern, with substantial variation in reported incidence across countries and emergency systems. According to the 2026 American Heart Association statistical update, 2024 data from the Cardiac Arrest Registry to Enhance Survival (CARES) showed an incidence of 78.7 EMS-treated OHCA cases per 100,000 inhabitants among people of all ages in the United States.^1^ Similarly, data from 15 national and regional registries participating in the International Liaison Committee on Resuscitation (ILCOR) indicated that the annual incidence of EMS-treated OHCA ranged from 30.0 to 100.2 cases per 100,000 population between 2015 and 2017.^2^ By comparison, the Out-of-Hospital Spanish Cardiac Arrest Registry reported an incidence of 24.2 cases per 100,000 population in 2022.^3^ Previous systematic reviews have likewise demonstrated considerable international heterogeneity in OHCA incidence.^4^ This variability likely reflects differences not only in population-level risk but also in case definitions, registry coverage, case-ascertainment methods, and whether emergency systems include dispatch-suspected, EMS-attended, or EMS-treated events.

Robust national registries are therefore essential for monitoring incidence, evaluating emergency medical services (EMS) performance, identifying regional risk patterns and guiding cardiovascular prevention and chain-of-survival policies.^5^, ^6^, ^7^ However, such surveillance remains limited in low- and middle-income countries (LMICs), where differences in demographic profiles, health-system access and reporting capacity restrict direct extrapolation from high-income settings. Available LMIC evidence reports OHCA or sudden cardiac death incidence ranging from 19.9 to 190 cases per 100,000 person-years.^8^ Latin America is particularly under-represented, one of the few identified studies, conducted in Medellín, Colombia, reported rates of 28.1 and 26.9 per 100,000 inhabitants in 2018 and 2019, respectively.^9^ No national study has described OHCA incidence or its temporal and geographical patterns in Ecuador.

To address the critical gap in national-level data on OHCA in Latin America, the present study aims to determine the incidence of out-of-hospital cardiac arrest in Ecuador, analyse its temporal trends over the study period, and explore ecological correlations with cardiovascular risk factors and socioeconomic indicators. Establishing a foundational epidemiological baseline will be key for informing future policy and improving the chain of survival in the region.

## Methods

### Study design and settings

In accordance with STROBE guidelines,^10^ we conducted a retrospective observational study using emergency report data collected by the Integrated Security Service (SIS ECU 9-1-1) from 1 January 2022 to 31 December 2024. Under Ecuadorian regulations, SIS ECU 9-1-1 databases are public, fully anonymised and available for research through the national open-data platform, “*Datos Abiertos Ecuador*”.^11^, ^12^

Ecuador is located in north-western South America and has a population of approximately 18 million. The country is undergoing an epidemiological transition characterised by population ageing, urbanisation and increasing prevalence of chronic non-communicable diseases. Ischaemic heart disease is the leading cause of death, accounting for 15.2% of deaths reported in 2023. Official data also describe relevant prevalences of hypertension (23%), diabetes (6.4%), obesity (35%), sedentary behaviour (52%) and smoking (12%), all of which contribute to cardiovascular risk.^13^, ^14^

SIS ECU 9-1-1 is the national entity responsible for receiving and coordinating emergency calls related to health, security and disasters. Calls to the unified emergency number are triaged and routed to the appropriate dispatch console. For health emergencies, licenced prehospital evaluators identify the primary cause, determine severity and coordinate response units.^15^

Basic Life Support ambulances are staffed by a driver and a paramedic, whereas Advanced Life Support ambulances include a driver and two paramedics.^16^ These units provide prehospital care and transport patients to an appropriate medical facility, and the resulting service record is stored in the national emergency database.

### Study population and variables

The SIS ECU 9-1-1 database centralises all incidents reported across Ecuador. We extracted records from 1 January 2022 to 31 December 2024. Security-related events and traffic accidents were excluded to focus exclusively on health-related incidents.

Within the medical emergency classification, incidents are categorised as clinical, traumatic, psychiatric, obstetric-gynaecological or cardiac arrest, among others. For this study, OHCA was defined as an out-of-hospital event recorded through the 9-1-1 emergency system and confirmed on scene by the responding prehospital team after verification of pulselessness and electrocardiographic rhythm assessment. Cases were therefore not included solely on the basis of dispatch suspicion. When an incident was initially dispatched under another medical emergency category, but cardiac arrest was identified or witnessed by EMS personnel after arrival, the final service record was classified as OHCA and included. Traumatic OHCA was excluded because these events are recorded under a separate traumatic-event code. Records with incomplete or missing fields were excluded. Because of the structure of the dataset, patient age, sex, initial rhythm, treatment and outcome variables were unavailable and could not be analysed.

All available records were exported to Excel, tabulated and organised by temporal distribution (day, month and year), geographical distribution (province) and frequency. This structure allowed assessment of spatial and temporal variability and identification of potential seasonal or regional patterns.

To ensure data quality, a three-stage review process was implemented. Three researchers, including two with master's degrees in public health and the principal investigator, independently reviewed the dataset for consistency and accuracy. Discrepancies were resolved through inter-observer reconciliation until consensus was reached.

### Statistical analysis

Descriptive statistics were used to present frequencies and annual OHCA incidence rates. Temporal trends were visualised with line charts, and geographical patterns were represented using heat maps. Incidence rates were calculated per 100,000 inhabitants using national population estimates by year and province. Annual incidence rates were calculated by dividing the number of recorded OHCA events by the corresponding national-year population estimate and multiplying the result by 100,000; the 95% confidence intervals were also calculated based on a Poisson distribution.

Statistical analysis was conducted using R Statistical Software version 4.5.0, which enabled calculation of frequency distributions, incidence rates and correlation analyses. Scientific visualisation was performed with SCImago Graphica Beta 1.0.48.

To assess possible structural or clinical determinants of OHCA variability across provinces, we explored ecological associations between OHCA incidence and hypertension, diabetes mellitus, acute myocardial infarction and provincial gross value added (GVA) per capita. These variables were selected because they represent major cardiovascular conditions, chronic diseases, causes of mortality and socioeconomic indicators in Ecuador during the study period.^13^, ^14^ Spearman's rank-order correlation coefficient (rho) was used to assess monotonic relationships using province-year aggregated data. No patient-level associations were calculated.

Quality control measures were applied during tabulation and analysis to minimise interpretation bias and assess the consistency of observed trends. In accordance with international ethical standards and local regulatory frameworks,^17^ the study was reviewed and approved by the Institutional Review Board of Universidad UTE, which waived informed consent because only publicly available, fully anonymised data were used (CEISH-UTE01-2026-004; January 2026).

## Results

During the study period, SIS ECU 9-1-1 recorded 936,784 emergency events, of which 1.06% (*n* = 9942) were confirmed and classified as EMS-attended OHCA. The year 2022 accounted for 36.56% (*n* = 3635) of all cases, with January showing the highest single-month count across the study period (392 cases).

**Fig. 1:**
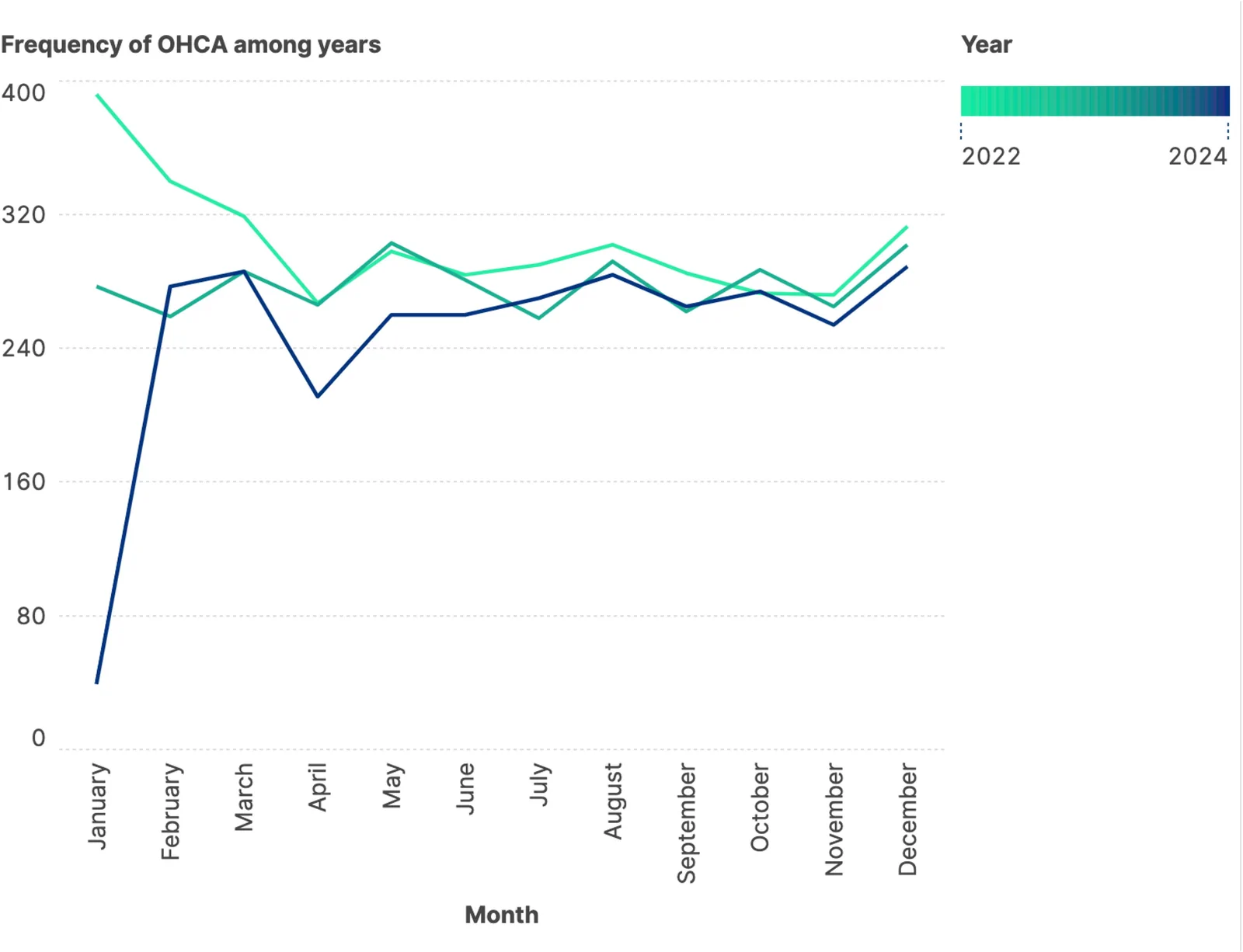
**Temporal trends of out-of-hospital cardiac arrest (OHCA) in Ecuador, 2022–2024**. The figure shows annual confirmed EMS-attended OHCA case counts and temporal trends over the study period.

Annual OHCA counts declined from 3635 in 2022 to 3338 in 2023 (−8.2%) and 2969 in 2024 (−11.0% versus 2023). After adjustment by official population estimates from the Instituto Nacional de Estadística y Censos (INEC),^18^ incidence rates were 20.51(95% CI: 19.85–21.19), 18.71(95% CI: 18.08–19.36) and 16.52 (95% CI: 15.93–17.13) per 100,000 inhabitants in 2022, 2023 and 2024, respectively (Fig. 1**).**

Monthly distributions showed reproducible but modest seasonality (Table 1). In 2022, monthly proportions ranged from 7.3% in April to 10.8% in January, clustering between 8% and 10% for most months. The 2023 pattern was similarly compact (7.7–9.1%), with subtle peaks in May, August and December. In 2024, January presented an anomalously low count (39 cases; 1.3%), after which monthly proportions returned to the usual 8–10% range and peaked again in December (289 cases; 9.7%).

**Table 1:** Monthly frequency of out-of-hospital cardiac arrest (OHCA) in Ecuador.

	**Year**	**2022**			**2023**			**2024**		
	**Month**	**Frequency**	**% Total**	**% Accumulated**	**Frequency**	**% Total**	**% Accumulated**	**Frequency**	**% Total**	**% Accumulated**
Out-of-hospital cardiac arrest	January	392	10.8 %	10.8 %	277	8.3 %	8.3 %	39	1.3 %	1.3 %
	February	340	9.4 %	20.1 %	259	7.8 %	16.1 %	277	9.3 %	10.6 %
	March	319	8.8 %	28.9 %	286	8.6 %	24.6 %	286	9.6 %	20.3 %
	April	267	7.3 %	36.3 %	266	8.0%	32.6 %	211	7.1 %	27.4 %
	May	298	8.2 %	44.5 %	303	9.1 %	41.7 %	260	8.8 %	36.1 %
	June	284	7.8 %	52.3 %	281	8.4 %	50.1 %	260	8.8 %	44.9 %
	July	290	8.0 %	60.2 %	258	7.7 %	57.8 %	270	9.1 %	54.0 %
	August	302	8.3 %	68.6 %	292	8.7 %	66.6 %	284	9.6 %	63.6 %
	September	285	7.8 %	76.4 %	262	7.8 %	74.4 %	265	8.9 %	72.5 %
	October	273	7.5 %	83.9 %	287	8.6 %	83.0%	274	9.2 %	81.7 %
	November	272	7.5 %	91.4 %	265	7.9 %	91.0 %	254	8.6 %	90.3 %
	December	313	8.6 %	100 %	302	9.0 %	100 %	289	9.7 %	100%

Cumulative data showed that, in all three years, more than half of the annual burden was reached between June and July. Regardless of inter-annual variability December consistently ranked among the highest-incidence months, suggesting a relevant period for emergency preparedness. The sharp January 2024 trough warrants further evaluation to differentiate true epidemiological change from under-reporting or data-entry delay.

**Fig. 2:**
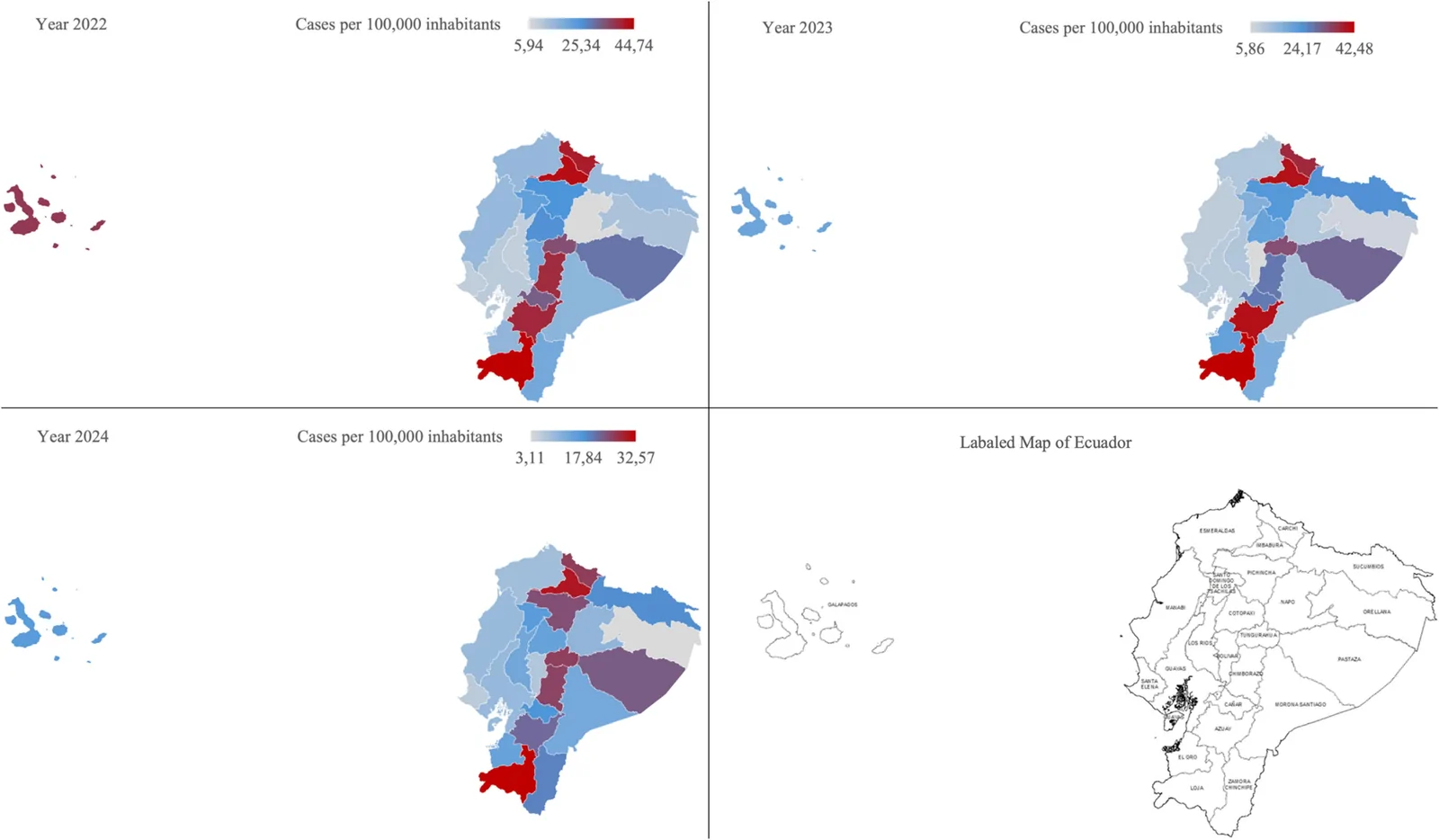
**Heat map of temporal and geographical trends of out-of-hospital cardiac arrest (OHCA) in Ecuador by province and year**. Incidence rates are expressed per 100,000 inhabitants. The grey spectrum indicates lower incidence, while the darker the spectrum, up to red, the higher the incidence. (For interpretation of the references to colour in this figure legend, the reader is referred to the web version of this article.)

Geographical analysis showed substantial variability across provinces and over time (Fig. 2). In 2022, the highest adjusted incidence rates were observed in Loja (44.74 per 100,000), Imbabura (43.01) and Carchi (40.93). These provinces remained among the highest across the three years, although their rates declined progressively, mirroring the national trend (Table 2).

**Table 2:** Geographical distribution of out-of-hospital cardiac arrest (OHCA) in Ecuador by province and year.

**Year**	**2022**				**2023**				**2024**			
**Province**	**Frequency**	**Population**	**Incidence rate/100,000**	**% Total**	**Frequency**	**Population**	**Incidence rate/100,000**	**% Total**	**Frequency**	**Population**	**Incidence rate/100,000**	**% Total**
Azuay	326	826,246	39.46	9.0 %	334	828,172	40.33	10.0 %	184	831,22	22.14	6.2 %
Bolivar	40	205,544	19.46	1.1 %	12	204,819	5.86	0.4 %	15	204,41	7.34	0.5 %
Cañar	77	237,584	32.41	2.1 %	65	237,527	27.37	1.9 %	42	237,47	17.69	1.4 %
Carchi	74	180,776	40.93	2.0 %	67	179,142	37.4	2.0 %	47	177,813	26.43	1.6 %
Chimborazo	193	493,084	39.14	5.3 %	136	490,774	27.71	4.1 %	126	488,857	25.77	4.2 %
Cotopaxi	124	480,27	25.82	3.4 %	100	484,792	20.63	3.0 %	74	489,147	15.13	2.5 %
El Oro	120	741,473	16.18	3.3 %	158	744,811	21.21	4.7 %	113	748,627	15.09	3.8 %
Esmeraldas	92	595,674	15.44	2.5 %	67	59,843	11.2	2.0 %	55	601,626	9.14	1.9 %
Galapagos	11	29,463	37.33	0.3 %	6	29,808	20.13	0.2 %	5	30,082	16.62	0.2 %
Guayas	480	4,643,432	10.34	13.2 %	478	4,690,613	10.19	14.3 %	485	4,739,771	10.23	16.3 %
Imbabura	209	485,913	43.01	5.7 %	197	489,961	40.21	5.9 %	150	494,035	30.36	5.1 %
Loja	222	496,194	44.74	6.1 %	211	496,685	42.48	6.3 %	162	497,438	32.57	5.5 %
Los Rios	89	953,06	9.34	2.4 %	108	959,805	11.25	3.2 %	135	968,66	13.94	4.5 %
Manabi	253	1,670,700	15.14	7.0 %	160	1,684,317	9.5	4.8 %	166	1,699,434	9.77	5.6 %
Morona Santiago	33	199,453	16.55	0.9 %	25	202,851	12.32	0.7 %	28	206,327	13.57	0.9 %
Napo	8	134,768	5.94	0.2 %	15	136,622	10.98	0.4 %	15	138,541	10.83	0.5 %
Orellana	23	186,166	12.35	0.6 %	14	189,568	7.39	0.4 %	6	192,831	3.11	0.2 %
Pastaza	34	114,969	29.57	0.9 %	35	117,062	29.9	1.0 %	28	119,032	23.52	0.9 %
Pichincha	821	3,231,547	25.41	22.6 %	754	3,250,537	23.2	22.6 %	807	3,272,265	24.66	27.2 %
Santa Elena	38	399,185	9.52	1.0 %	43	400,829	10.73	1.3 %	26	403,478	6.44	0.9 %
Santo Domingo de los Tsachilas	116	513,692	22.58	3.2 %	90	518,576	17.36	2.7 %	87	523,524	16.62	2.9 %
Sucumbios	31	207,221	14.96	0.9 %	51	206,36	24.71	1.5 %	38	205,253	18.51	1.3 %
Tungurahua	197	573,786	34.33	5.4 %	189	576,407	32.79	5.7 %	152	579,082	26.25	5.1 %
Zamora Chinchipe	23	115,101	19.98	0.6 %	21	116,363	18.05	0.6 %	23	117,65	19.55	0.8 %

In absolute terms, Pichincha consistently reported the largest number of OHCA cases, accounting for 22.6% in 2022, 22.6% in 2023 and 27.2% in 2024. However, its population-adjusted incidence remained moderate, ranging from 23.20 to 25.41 per 100,000. Conversely, Guayas, despite being the most populous province, showed one of the lowest adjusted rates, around 10.2 per 100,000 across the three years, suggesting either lower recorded risk or possible under-reporting.

Most provinces showed a reduction in OHCA incidence between 2022 and 2024. Azuay decreased from 39.46 to 22.14 per 100,000, while Tungurahua decreased from 34.33 to 26.25. Los Ríos diverged from this pattern, increasing from 9.34 in 2022 to 13.94 per 100,000 in 2024, warranting further local investigation.

Provincial contributions to the total national OHCA burden remained relatively stable for most territories. However, smaller provinces such as Galápagos, Napo, and Zamora Chinchipe reported low case numbers and population-adjusted rates, contributing under 1% of national cases annually.

**Fig. 3:**
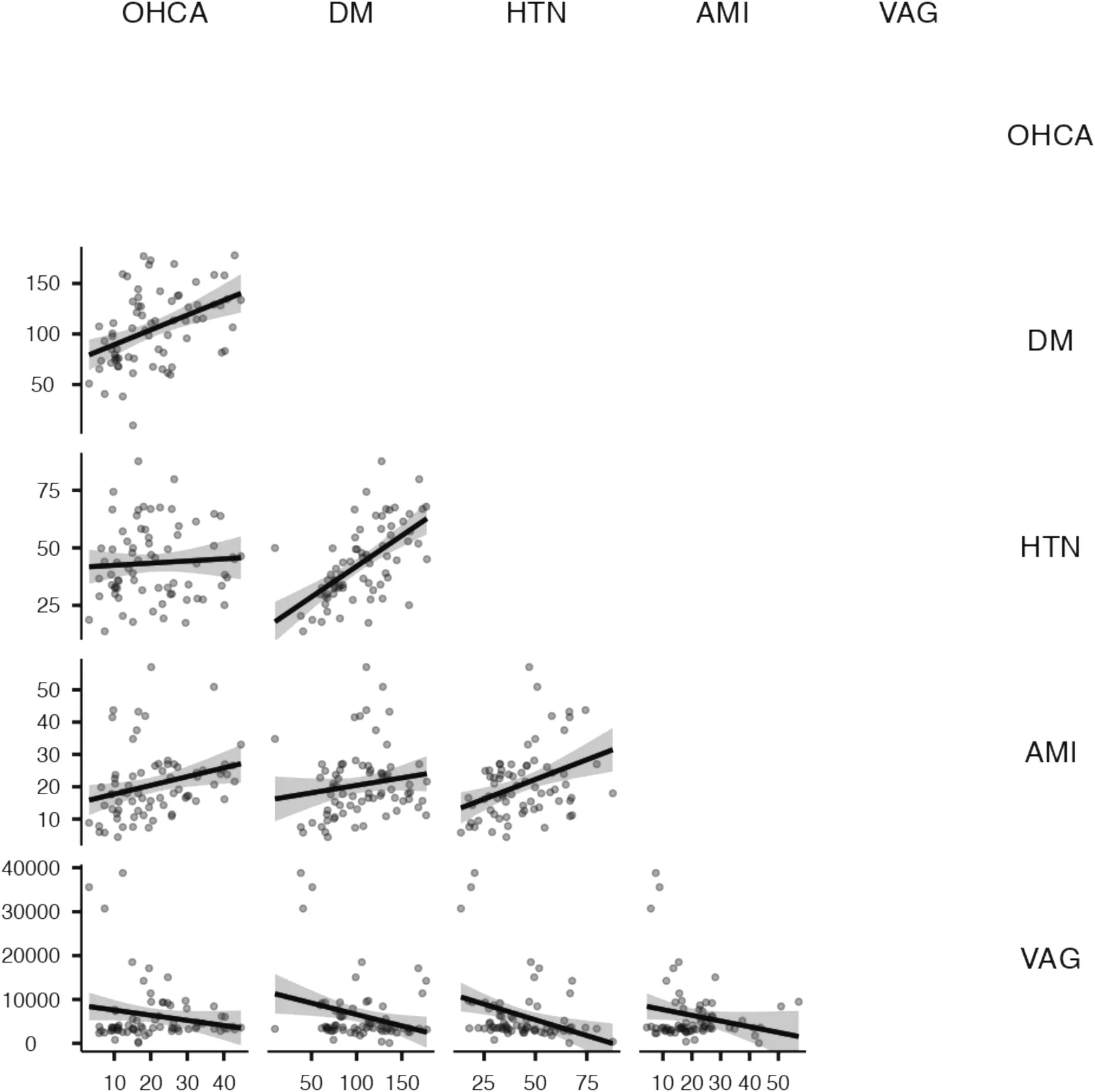
**Spearman scatterplots between out-of-hospital cardiac arrest (OHCA) incidence and provincial comorbidities/economic indicators**. DM = diabetes mellitus; HTN = hypertension; AMI = acute myocardial infarction; GVA = Gross Value Added. All correlations are ecological and based on province-year aggregated data.

Using province-year aggregated data, we explored ecological correlations between OHCA incidence, prevalent comorbidities such as hypertension (HTN), diabetes mellitus (DM), and acute myocardial infarction (AMI) and Gross Value Added (GVA) per capita using Spearman’s correlation (Table 3**;** Fig. 3). OHCA showed a moderate and statistically significant correlation with diabetes mellitus (rho = 0.463; 95% CI, 0.249–0.636; *p* < 0.001), while its association with hypertension was weak and non-significant (rho = 0.075; 95% CI, −0.160 to 0.302; *p* = 0.531). A weak but statistically significant correlation was found with acute myocardial infarction (rho = 0.382; 95% CI, 0.157–0.570; *p* < 0.001). Hypertension and diabetes were strongly correlated (rho = 0.653; 95% CI, 0.478–0.778; *p* < 0.001). GVA per capita showed a weak, non-significant correlation with OHCA (rho = 0.060; 95% CI, −0.174 to 0.288; *p* = 0.614), indicating that provincial economic output alone did not explain regional variability. Because these analyses were ecological, the observed associations should be interpreted as province-level patterns rather than individual patient-level risk estimates.

**Table 3:** Ecological province-year distribution of OHCA incidence, prevalent comorbidities, and economic indicators.

**Year**	**2022**					**2023**					**2024**				
**Province**	**OHCA per 100,000**	**GVA per Capita**	**HTN per 100,000**	**DM per 100,000**	**AMI per 100,000**	**OHCA per 100,000**	**GVA per Capita**	**HTN per 100,000**	**DM per 100,000**	**AMI per 100,000**	**OHCA per 100,000**	**GVA per Capita**	**HTN per 100,000**	**DM per 100,000**	**AMI per 100,000**
Azuay	39.46	$6153	33.53	81.57	25.17	40.33	$6042	38.28	83.07	26.93	22.14	$6248	32.6	84.82	27.07
Bolivar	19.46	$2307	54.49	98.28	7.30	5.86	$2317	36.62	107.41	7.81	7.34	$2433	44.03	92.95	14.19
Cañar	32.41	$3434	61.45	151.53	15.57	27.37	$3400	55.57	137.67	26.10	17.69	$3459	31.58	118.33	16.42
Carchi	40.93	$3319	37.06	134.42	23.79	37.40	$3307	64.75	158.53	20.65	26.43	$3337	79.86	169.28	26.99
Chimborazo	39.14	$2814	63.88	128.17	23.93	27.71	$2765	59.5	138.35	19.15	25.77	$2785	66.89	132.55	10.84
Cotopaxi	25.82	$3498	29.98	67.05	11.45	20.63	$3493	22.28	67.45	9.49	15.13	$3427	17.79	61.13	7.56
El Oro	16.18	$5154	64.06	121.25	37.49	21.21	$5386	45.65	112.91	26.45	15.09	$5567	39.00	132.11	23.24
Esmeraldas	15.44	$3065	35.93	75.71	10.58	11.2	$3017	28.24	67.68	12.70	9.14	$2835	38.4	71.31	17.62
Galapagos	37.33	$8434	50.91	128.98	50.91	20.13	$9464	46.97	110.71	57.03	16.62	$7400	66.48	136.29	43.22
Guayas	10.34	$7346	33.04	76.69	23.69	10.19	$7656	32.51	79.73	22.53	10.23	$7830	29.96	74.27	20.97
Imbabura	43.01	$3149	45.07	177.81	21.61	40.21	$3379	25.10	157.97	16.12	30.36	$3229	34.01	126.31	17.21
Loja	44.74	$3584	46.35	133.62	33.05	42.48	$3806	45.9	106.51	26.58	32.57	$3838	43.22	114.39	24.32
Los Rios	9.34	$3663	33.89	84.67	17.73	11.25	$4069	35.63	75.95	15.32	13.94	$4114	40.98	77.12	20.34
Manabi	15.14	$3290	49.92	90.46	34.78	9.50	$3505	66.67	97.67	41.5	9.77	$3567	74.38	110.74	43.72
Morona Santiago	16.55	$2427	44.62	144.39	12.53	12.32	$2581	57.18	159.23	18.24	13.57	$2561	52.83	157.03	17.93
Napo	5.94	$3802	28.94	65.3	5.94	10.98	$3621	35.87	68.07	4.39	10.83	$3615	32.48	75.07	10.11
Orellana	12.35	$38,819	20.41	38.14	7.52	7.39	$30,716	13.72	40.62	5.80	3.11	$35,585	18.67	50.82	8.82
Pastaza	29.57	$9703	17.40	113.07	16.53	29.90	$7943	27.34	95.68	17.08	23.52	$9339	19.32	81.49	14.28
Pichincha	25.41	$8671	29.80	59.6	22.81	23.20	$9325	25.57	65.16	24.89	24.66	$9185	32.73	61.52	26.98
Santa Elena	9.52	$2998	49.35	100.45	13.03	10.73	$3037	43.66	84.82	11.73	6.44	$2852	49.82	73.36	19.83
Santo Domingo De Los Tsachilas	22.58	$3704	67.55	142.3	16.55	17.36	$3863	58.24	127.27	24.3	16.62	$3880	87.68	127.6	17.96
Sucumbios	14.96	$18,501	47.78	105.68	15.44	24.71	$15,045	49.43	98.86	28.11	18.51	$1771	57.98	103.77	41.9
Tungurahua	34.33	$4511	27.54	115.37	25.10	32.79	$4776	27.93	128.73	23.07	26.25	$4705	34.71	113.28	17.96
Zamora Chinchipe	19.98	$11,406	66.90	172.89	15.64	18.05	$14,238	67.89	177.03	11.17	19.55	$17,108	51.85	168.3	13.6

## Discussion

This study provides the first national-level epidemiological description of confirmed EMS-attended OHCA in Ecuador and contributes to the limited evidence available from Latin America. The main findings were a progressive decline in national incidence between 2022 and 2024, modest seasonal variation, substantial geographical heterogeneity and relevant ecological associations with diabetes mellitus and acute myocardial infarction.

A central methodological consideration is that this study estimates confirmed EMS-attended OHCA recorded in the national emergency system, rather than all sudden deaths in the community, all dispatch-suspected cardiac arrests or all resuscitation-attempted cases. This distinction is important because international incidence estimates vary substantially according to registry inclusion criteria, confirmation processes and whether cases are restricted to presumed cardiac aetiology. It also clarifies that the study should be interpreted as a surveillance-based national profile rather than a complete measure of all community cardiac deaths.

The incidence observed in Ecuador (16.5–20.5 per 100,000 inhabitants) is therefore lower than the 2024 CARES estimate of 78.7 per 100,000 and the 30.0–100.2 range reported by ILCOR registries.^1^, ^2^ Direct comparison is limited by differences in case definitions, case ascertainment and registry maturity. The SIS ECU 9-1-1 database is an administrative dispatch and response dataset rather than a dedicated Utstein-style OHCA registry; consequently, events not reported through 9-1-1, arrests in underserved areas and misclassified records may not have been captured. Nevertheless, the Ecuadorian estimate is near the lower end of the broad range reported in LMICs (19.9–190 per 100,000 inhabitants) and is closer to the city-based rates reported in Medellín, Colombia (28.1 and 26.9 per 100,000 inhabitants).^8^, ^9^ This variability reinforces the need for country-specific surveillance and cautious cross-national interpretation.

The observed decline from 20.5 to 16.5 cases per 100,000 inhabitants aligns with the variable temporal patterns described in the literature. Alrawashdeh et al. reported annual reductions of up to 7%, whereas McBride et al. found no evidence of a linear temporal change in overall incidence and showed that temporal patterns differed according to demographic and geographic characteristics, and longer-term national data have also documented declining trends.^19^, ^20^, ^21^ Given the three-year observation period, our finding should be interpreted as a short-term decline in recorded incidence rather than evidence of a sustained epidemiological transition. Changes in cardiovascular prevention, access to care or EMS performance may have contributed, but causality cannot be established and temporal variation in case ascertainment or reporting remains possible.^22^, ^23^

The seasonal pattern, with December and January among higher-incidence periods, is consistent with international literature describing higher cardiac arrest incidence during winter months in temperate regions, potentially related to cold exposure, respiratory infections and psychosocial stress.^24^ Ecuador's equatorial climate does not have marked winter conditions, so these fluctuations may instead be influenced by year-end behavioural changes, alcohol intake, reduced physical activity, dietary excess and circulation of influenza-like illness. Additionally, the circulation of influenza-like illness may underlie these fluctuations, consistent with prior studies linking respiratory infections to cardiovascular events.^25^

The geographical disparities observed across Ecuador are particularly relevant. Loja, Imbabura and Carchi consistently reported high adjusted incidence. These provinces share high-altitude geography (>2000 m above sea level), rural dispersion and potential limitations in EMS coverage. This finding contrasts with studies suggesting that high-altitude exposure may have protective cardiovascular effects through adaptive mechanisms.^26^, ^27^ Therefore, the observed pattern may be more strongly influenced by service access, rurality, emergency response times and local reporting practices. Previous OHCA research has shown that rurality and area deprivation are associated with worse outcomes after cardiac arrest.^28^

In contrast, Guayas, the most populous province, showed persistently low adjusted incidence. This could reflect better healthcare infrastructure and urban EMS coverage, although under-reporting or differences in data capture cannot be excluded. Pichincha accounted for the largest absolute number of cases but had only moderate adjusted rates, consistent with other urban settings in which absolute burden follows population size while adjusted rates are shaped by access to care, EMS availability and public health resources.^29^, ^30^ The increase in Los Ríos – from 9.3 to 13.9 cases per 100,000 between 2022 and 2024 – diverged from the national pattern and may reflect local demographic change, rapid urbanisation, ageing or limited access to primary care, factors described in other temporal analyses.^31^, ^32^

The moderate ecological correlation between OHCA and diabetes mellitus observed aligns with studies that shows diabetes increases the risk (OR 1.6–1.8) of sudden cardiac death and cardiac arrest. Proposed mechanisms include autonomic dysfunction, accelerated atherosclerosis, microvascular ischaemia, pro-arrhythmic remodelling and hypoglycaemia-related arrhythmias.^33^, ^34^, ^35^

Although hypertension is a well-established cardiovascular risk factor, its province-level association with OHCA was weak and not statistically significant.^36^, ^37^ This may reflect heterogeneity in diagnosis, treatment coverage and blood-pressure control, as well as the coexistence of obesity and diabetes, which can dilute area-level correlations. The strong correlation between hypertension and diabetes in our data reinforces their frequent clustering as components of metabolic risk.^38^

The weak but significant correlation between OHCA and acute myocardial infarction aligns with the known pathophysiological link between acute coronary syndromes and cardiac arrest. International data indicate that 50–70% of OHCA patients present with significant coronary artery disease and that 8–10% of acute myocardial infarctions are complicated by prehospital cardiac arrest.^39^, ^40^ This overlap highlights the importance of early recognition, reperfusion pathways and secondary prevention.

Interestingly, absence of a significant association between GVA per capita and OHCA suggests that broad provincial economic indicators may not capture the social determinants most relevant to cardiac arrest. Evidence from Europe and North America indicates that neighbourhood deprivation, rather than regional economic averages, is more closely associated with OHCA incidence, bystander cardiopulmonary resuscitation and survival.^41^, ^42^, ^43^, ^44^ Local factors such as health literacy, CPR training, AED distribution and EMS response times may therefore better explain OHCA variation.

Overall, these findings underscore spatial and temporal disparities in OHCA incidence in Ecuador. They support region-specific strategies that integrate cardiovascular prevention, rapid EMS activation, equitable access to acute care, community CPR training and broader AED deployment, particularly in provinces with persistent or increasing incidence. Importantly, these actions should be adapted to local EMS capacity, geography and reporting practices rather than implemented as uniform national measures. Provinces with high adjusted rates may require different interventions from highly populated provinces with greater absolute case numbers, because prevention, preparedness and resource allocation respond to different epidemiological signals.

### Limitations

These findings should be interpreted in light of several limitations. The administrative database did not include patient-level demographic, clinical, rhythm, treatment, response-time or outcome data, preventing survival analysis, risk stratification, age-adjusted comparisons and Utstein-style indicators.^45^ Although OHCA classification required both emergency call registration and prehospital confirmation, residual under-reporting or misclassification cannot be excluded, especially in remote or underserved areas. EMS-witnessed arrests could be captured when the final service record was classified as OHCA, but the dataset did not contain a witness-status variable; therefore, the completeness of this subgroup could not be independently verified. Correlation analyses were ecological and based on province-year aggregates; therefore, associations with comorbidities, acute myocardial infarction and GVA should not be interpreted as individual-level or causal relationships. In addition, differences in provincial reporting practices may have influenced apparent geographical variation, particularly where EMS access or data feedback processes differ between urban and rural areas. Nevertheless, the use of national data and the consistency of several temporal and regional trends provide a useful baseline for future research.

### Implications for practice and future research

Future efforts should focus on developing a dedicated national OHCA registry aligned with Utstein reporting standards.^45^ Such a registry would allow monitoring of outcomes, identification of at-risk populations and evaluation of interventions. It should also include variables unavailable in the present database, such as age, sex, initial rhythm, response intervals, bystander CPR, defibrillation, treatment and survival. Public health initiatives should prioritise provinces with persistently high or increasing incidence, expand community CPR training and AED access, and strengthen EMS coverage in rural and high-altitude areas. Linking registry data with hospital outcomes would further support quality improvement and policy evaluation.

## Conclusion

OHCA incidence in Ecuador decreased progressively between 2022 and 2024, with modest seasonal patterns and marked regional heterogeneity. Diabetes mellitus and acute myocardial infarction showed a relevant ecological correlation. These findings provide a national epidemiological baseline and support the establishment of an OHCA registry to guide targeted prevention, emergency planning, resource allocation, EMS strengthening and policy development in Ecuador and comparable Latin American contexts.

## CRediT authorship contribution statement

**Ricardo S. Pinto-Villalba:** Writing – review & editing, Writing – original draft, Visualization, Validation, Supervision, Software, Resources, Project administration, Methodology, Investigation, Funding acquisition, Formal analysis, Data curation, Conceptualization. **Mónica Martínez:** Writing – review & editing, Writing – original draft, Validation, Investigation, Formal analysis, Conceptualization. **Carlos Guerrero-Calderón:** Writing – review & editing, Writing – original draft, Visualization, Software, Investigation, Conceptualization. **Jefferson Reinoso:** Writing – review & editing, Writing – original draft, Investigation, Conceptualization. **Carla Zamora:** Writing – review & editing, Writing – original draft, Investigation, Conceptualization. **Rene M. Abarca-Tenemasa:** Writing – review & editing, Writing – original draft, Visualization, Validation, Supervision, Resources, Project administration, Investigation, Funding acquisition, Conceptualization.

## Ethics approval

This study was reviewed and approved by the Institutional Review Board (IRB) of Universidad UTE. The IRB granted approval and waived the requirement for informed consent (IRB number: CEISH-UTE01-2026-004; approval date: January 2026).

## Funding sources

The author(s) declare that financial support was received for the research and/or publication of this article. Publication of this article was funded by Universidad UTE.

## Declaration of competing interest

The authors declare that the research was conducted in the absence of any commercial or financial relationships that could be construed as a potential conflict of interest.
